# Traumatic Brain Injury in United States Operation Enduring Freedom/Operation Iraqi Freedom (OEF/OIF) Hispanic Veterans—A Review Using the PRISMA Method

**DOI:** 10.3390/bs6010003

**Published:** 2016-01-12

**Authors:** Vanessa D. Arriola, Jeffrey W. Rozelle

**Affiliations:** Department of Epidemiology, School of Public Health and Tropical Medicine, Tulane University, 1440 Canal Street, New Orleans, LA 70112, USA; jrozelle@tulane.edu

**Keywords:** traumatic brain injury, TBI, Hispanic, veteran

## Abstract

Traumatic brain injury (TBI) is commonly defined by Menon *et al.* as an “alteration of the brain function, or other evidence of brain pathology, caused by an external force.” TBI can be caused by penetrating trauma to the head in which the magnitude of the injury is dependent on the magnitude of the forces that are applied to the head. The consequences of TBI can range from minimal to severe disability and even death. The major objectives of this systematic review are to survey the current literature on Operation Enduring Freedom (OEF) and Operation Iraqi Freedom (OIF) Hispanic veterans with TBI. To complete this analysis, the Preferred Reporting Items for Systematic Reviews and MetaAnalysis (PRISMA) identified 875 articles in common and retrieved a total of 34 articles that met the inclusion criteria, consisted of OEF/OIF Hispanic veterans, reported quantitative data, and were conducted with adult U.S. veterans living in the United States. Since TBI diagnosis was unclear in most articles, only five articles that used the VATBIST instrument were analyzed. The results suggested that there is a lack of research on OEF/OIF Hispanic veterans and Hispanic subgroups. Future studies need to be conducted to consider minority groups while analyzing data involving TBI.

## 1. Background and Significance

Traumatic brain injury (TBI) has been denoted the “signature wound” of the Iraq and Afghanistan wars [1]. Often, TBI is the result of an impact to the head or a penetrating trauma. Other times, TBI occurs without any visible wounds, such as when explosives strike the head in combat situations [2]. United States military personnel are believed to be at a higher risk to incur TBIs. These are usually mild, but can create serious and persistent sequelae [3]. Certain mental health disorders are linked with mild TBI including depression, substance-abuse, self-destructive behavior, and post-traumatic stress disorder (PTSD). A recent survey of Operation Enduring Freedom (OEF) and Operation Iraqi Freedom (OIF) troops found that 11% of women and 20% of men tested positive for deployment-related TBI [3].

There are many recognized factors that contribute to health disparities. Research often examines the role of race, age, socioeconomic status, gender and more as determinants of health. Minority status and belonging to an underrepresented group is an important factor for long term outcomes of TBI and mTBI. Observational studies have found that women, for example, have significantly worse outcomes from TBI than men on various scales [4,5]. A retrospective study in 2007 compared whites and minorities in functional outcomes of traumatic brain injuries, and found more impaired performance among minorities [6]. After controlling for potential confounders, blacks and Hispanics fared significantly worse after one year compared to whites on the Disability Rating Scale, the Functional Independence Measure, and the Community Integration Questionnaire. Other studies have also found significantly worse outcomes among minorities compared to Caucasians on social outcomes such as employment [7,8]. Inquiry into these differences is important because it offers the information necessary to make crucial decisions and to develop effective interventions to reduce preventable morbidity and mortality [9,10,11,12,13].

There is a particular need to study Hispanic veterans since the number of minority veterans is increasing. According to recent statistics issued by the U.S. Department of Defense (DOD), Hispanics are currently 11.4% of the active-duty military forces, creating the second largest minority group serving the United States armed forces [2]. Hispanic servicemen and servicewomen are at an increased risk of mortality from TBI. Dismuke, Gebregziabher and Egede compared 14,690 veterans, 2.14% of which were Hispanics [14]. They found that being Hispanic (adjusted for race; HR = 2.3; 95% CI = 1.49–3.64) was strongly associated with a higher risk of mortality among veterans clinically diagnosed with TBI. Overall mortality at 48 months was 6.7% among Hispanics, compared to 2.9% in non-Hispanic whites, underscoring the need to study TBI in Hispanics. The Veterans Administration (VA) researchers are performing investigations with the purpose of improving the care of veterans with TBI. While Hispanic veterans have been the subject of many reports, most research has focused on PTSD. There has been little scientific investigation into the specific needs of Hispanic veterans with TBI.

### 1.1. Burden of TBI

Each year, TBI causes a substantial number of deaths and permanent disability according to the Centers for Disease and Control and Prevention (CDC). Roughly 5 million people in the US live with TBI related disability [15,16,17]. Since 2010, there have been approximately 2.5 million TBIs as from an isolated injury or along with other injuries [18]. Military members in particular are at a higher risk for TBI than the civilian population as a consequence of combat operations. According to a 2008 study, about 22% of active duty members who returned from OIF described incurring TBIs and concussions, and between 15 and 20% of OEF/OIF service members experienced mild TBI [19,20,21,22]. In 2013, a study found that 25% of screened veterans had probable TBI exposure, in which the majority of the exposures were blasts (~85%) [23].

Traumatic brain injury, however is not only limited to the battlefield. Between April 2007 to the end of the fiscal year in 2009, 66,023 veterans were found to have symptoms consistent with TBI through an outpatient screening of individuals receiving care at the VA after deployment in OEF or OIF [24]. While the incidence of diagnosed TBI has declined from 32,668 in 2011 to 18,564 in 2014 due to improved screening and surveillance efforts within the Military Health System, the cost of these injuries remain staggering [25,26]. TBIs cost the VA millions of dollars yearly in healthcare and other benefits.

In 2008, Veterans Health Administration (VHA) estimated that their services for OEF and OIF veterans cost between $121 billion and $285 billion [27]. Two years later, the Congressional Budget Office (CBO) projected that the 10 year spending by the VHA would reach $40 to $54 billion at $5170 per veteran per year. Current policies establish that veterans living with TBI are eligible for up to a 100% disability rating [28,29]. Considering this, understanding who TBI affects is essential in the coming years.

### 1.2. Definition of TBI

There is considerable variability across federal agencies regarding how TBI is classified. Some use symptoms such as loss of consciousness (LOC), alteration of consciousness (AOC), or posttraumatic amnesia (PTA), or they use the Glasgow Coma Scale (GCS). No consensus or standard exists regarding how these symptoms or scales should be used to define TBI severity. Most agencies include the concept that TBI is the effect of an unexpected trauma that causes harm to the brain [30]. The CDC defines it as any injury which is “caused by a bump, blow or jolt to the head or a penetrating head injury that disrupts the normal function of the brain” [18]. However, not all blows, jolts to the principal, or penetrating brain injuries cause traumatic brain trauma. The severity of TBI ranges from mild to severe [26,31], as shown in Table 1. According to the Defense and Veterans Brain Injury Center (DVBIC), the leading causes of TBI include falls, traffic accidents, assault, and being struck by or against an object [32]. Primary brain injury is typically categorized as focal (moderate to severe TBI), diffuse (mild injury), or mixed depending on the means of injury in the brain’s response [2].

**Table 1:** Severity of traumatic brain injury (TBI).

Severity	Glasgow Coma Scale Rating (GCS)	Loss of Consciousness (LOC)	Alteration of Consciousness (AOC)	Posttraumatic Amnesia (PTA)	Structural Brain Imaging
Mild	13–15	Up to 30 min	Up to 24 h	Up to 24 h	Normal
Moderate	9–12	30 min to 24 h	> 24 h	24 h to 7 days	Normal or abnormal
Severe	9–8	> 24 h	> 24 h	> 7 days	Normal or abnormal

Notes: Adapted from: [26].

Diagnosis of TBI involves imaging and questioning patients about their injury. Screening for symptoms stands as the best and fastest method to assess the possible exposure to an injury event to define if an AOC was apparent, if it was associated with the outcome, or if any neurologic changes of symptoms resulted from the event [24]. To this end, several actors including VA’s TBI healthcare providers, primary care providers DVBIC, and the Veteran’s Affairs Central Office collaborated to produce a questionnaire in 2006. The resulting Veteran Administration’s Traumatic Brain Injury Screening Instrument (VAT-BIST) is currently used to test military personnel after deployment [15]. The intent of the instrument is to identify veterans who had TBI-associated injuries and currently experienced TBI-associated symptoms. The instrument consists of four questions regarding exposure to a potential TBI event, loss or alteration of consciousness, and post-concussive symptoms at the time of the assessment. If a veteran presents a positive response to one element in each of the four questions, he/she will be considered to have possible TBI. If no element is identified, then the screening result is considered to be negative. In 2010, this instrument started to be used in all VA facilities to screen veterans who served after 11 September 2001 for TBI [33]. From 2007 to 2012, over 644,000 (95%) veterans were screened for TBI [34].

### 1.3. Comorbidities Associated with TBI

Emotional and behavioral assessments can help to acquire information from the veteran and/or family member or caregiver concerning comorbidities associated with TBI. Some of the most common comorbidities are suicidal ideation, depression, substance abuse, and PTSD. Suicidal ideation and depression are frequently seen in patients with brain injuries. A study found that 13.7% of OEF/OIF veterans with depression were diagnosed with TBI, and an increased risk of suicide was reported in those who had psychiatric comorbidities [21]. Furthermore, the RAND Corporation reported a prevalence of 19.5% probable TBI and 13.8% probable PTSD from a study of 1965 OEF/OIF veterans [21].

### 1.4. Surveillance of TBI Cases

CDC monitors cases of TBI-related injuries in the US general population. For the military population, the Armed Forces Health Surveillance Center collects TBI data using the Defense Medical Surveillance System and Theater Medical Data Store. Cases of TBI are defined based on MHS TBI coding guidance by the Unified Bio-statistical Utility working group [25]. For the veteran population, a roster of OEF/OIF veterans is maintained by the Department of Defense Manpower Data Center and the VA Office of Public Health, Post Deployment Health Group, Epidemiology Program. The DoD roster is connected to VA’s electronic inpatient and outpatient health records and collects health information using the standard International Classification of Diseases, 9th Revision, Clinical Modification (ICD-9CM) diagnostic codes [35]. The Office of Public Health at VHA reports quarterly on VA facility specific OEF/OIF and Operation New Dawn (OND) veterans diagnosed with potential or provisional PTSD and VA Health Care Utilization among OEF/OIF/OND.

## 2. Materials and Methods

Previous research and literature focused on the characteristics of Hispanics with post traumatic stress disorder (PTSD) and the significance of Hispanic culture on the diagnosis and treatment of PTSD. There is little literature that explicitly discusses the implications of traumatic brain injury (TBI) in OEF/OIF Hispanic veterans. To fill the gap, a review of the most relevant literature on Hispanic veterans with TBI was completed. To validate the review, the Preferred Reporting Items for Systematic Reviews and Meta-Analysis (PRISMA) was applied. The PRISMA Statement was created by 29 review authors, methodologists, clinicians, medical editors, and consumers. This statement contains a 27-items checklist and a four-phase flow diagram that is critical for transparent reporting of a systematic review [36].

### 2.1. Data Sources and Searches

Prior to starting the literature review, a search was made of previously published, ongoing systematic reviews, or meta-analyses of studies with the terms “Hispanic veteran” and “traumatic brain injury” at the Cochrane Library Online. No systematic reviews were found as of 19 January 2015. The second step consisted of a search for all medical subject headings of “Hispanic”, “traumatic brain injury” and “veteran” in the MeSH database available from PubMed’s homepage. Fourteen relevant terms were found for “Hispanic” and ten relevant terms were found for “traumatic brain injury”, but no relevant terms were found for “veteran”.

A search strategy was carefully developed with the assistance of the education librarians. All citations were exported to RefWorks database. A search was conducted in PubMed, EBSCO (MEDLINE, Psychology and Behavioral Sciences Collection, PsycTESTS, Rehabilitation Reference Center, CINAHL Plus), and an independent search was conducted for PsycINFO, Published International Literature on traumatic stress (PILOTS), LEXISNEXIS, and AMSUS (The Society of Federal Health Professionals) from 1980 to the date last searched (2 March 2015) limited to English language, peer reviewed publications, and human subjects. The search terms and MeSH headings included the following: veteran(s), Latino, Hispanic, Hispanic American, traumatic brain injury, TBI, Brain injury. Combined terms included Latino, Hispanic, veteran, traumatic brain injury, and TBI. Furthermore, a Microsoft Access 2010 database was created to organize material, remove duplicates that could not be removed by RefWorks, and enter new information relevant to the search. Additional articles and books from reference lists of relevant subjects and disciplines were found at the VA National Desktop Library. Finally, the author subscribed to weekly PubMed literature e-Alerts prepared by professional librarians in the VA library network to stay up-to-date on the latest developments in the follow selected fields, which included OEF/OIF veterans, traumatic brain injury, minority veterans, and veteran/military suicide.

Abstracts that met the following criteria were considered relevant: (a) concerned OEF/OIF Hispanic veterans; (b) reported quantitative data and was published on a peer-reviewed journal; (c) was conducted on an adult U.S. veteran living in the United States. Studies that were not conducted on adult OEF/OIF Hispanic veterans who lived in the United States and those that used only qualitative data were excluded.

### 2.2. Study Selection

One reviewer (V.A.) merged search results using reference management software. The software removed duplicate records of the same report. Two independent reviewers screened all titles and/or abstracts that met the inclusion criteria (V.A. and J.R.).

### 2.3. Data Extraction and Quality Assessment

**Figure 1:**
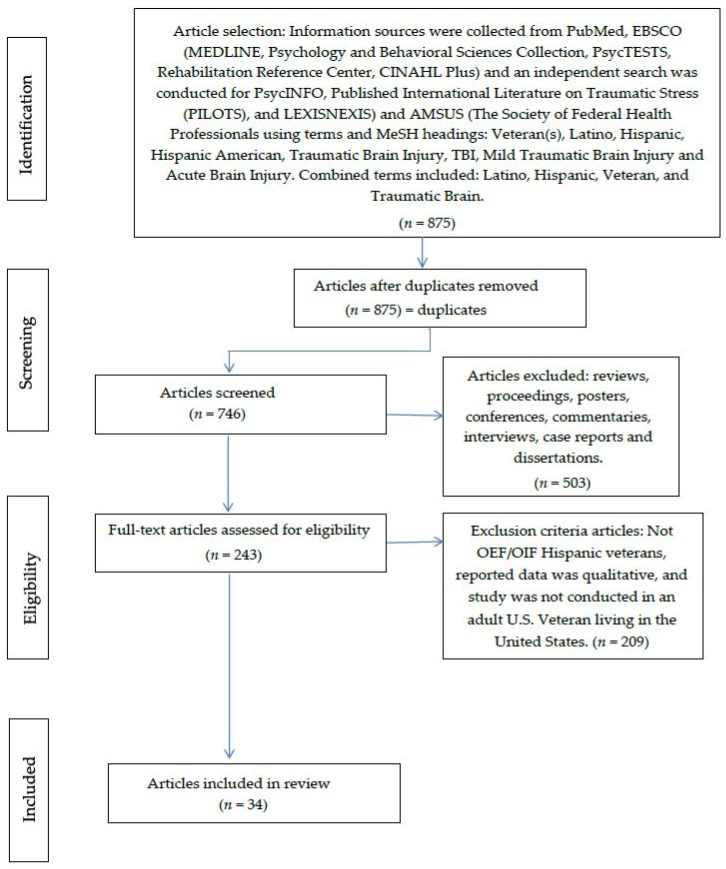
PRISMA flow diagram.

The initial query yielded 79,005 potential articles. With the search strategy in place, 875 abstracts were retrieved on 30 January 2015 by two reviewers. On the second search made at the end of February and beginning of March 2015, abstracts of peer-reviewed articles published from that time period were reviewed. After applying inclusion and exclusion criteria at the abstract level, 243 full-text articles were retrieved for further evaluation, as shown in Figure 1.

A complete search terms, strategy in detail, and results are described in Appendix A. 

## 3. Results

Since the VAT-BIST is the most accepted instrument to screen for TBI in military personnel, only the five articles that used this instrument were taken in consideration for this review. Twenty-nine out of thirty-four studies produced by the PRISMA method assessed veterans with TBI and PTSD, and did not use the VAT-BIST. Without the VAT-BIST, diagnosis of TBI was unclear. The remaining five articles studied veterans with TBI and their overall functional status, which included physical, cognitive and behavioral/emotional indicators such as post concussive symptoms, headaches, depression, memory and communication, as shown in Table 2. Total sample sizes ranged from 57 to 303, 716 (1.3% to 21.4%) Hispanic veterans. None of the studies included Latino subgroups or recorded the number or percentage of Hispanic veteran women (Latinas). All of the studies accessed data supplied by the VA or DOD, and three out of five studies targeted participants at the VA Polytrauma Center. All veterans were from the OEF/OIF Combat era and were less than 55 years old while in combat.

Two retrieved articles reported physical results (post-concussive symptoms and headaches) [37,38]. Ettenhofer *et al.* found that, from a sample of 57 (21.4% Hispanic), 34% of veterans with TBI self-reported post-concussive symptoms (from severe to very severe) [39]. Furthermore, Patil *et al.* found that the prevalence of headaches among veterans with MTBI was 20%, and 92% reported some level of sleep disturbances [40]. However, headache rating did not differ by ethnicity (Hispanic/Latino *vs*. non-Hispanic/Latino) or marital status (married *vs.* unmarried).

Two of the articles retrieved reported cognitive results (communication and visual/verbal learning and memory) [37,38]. Norman *et al.* reported that among 303,716 (~12% Hispanics veterans), prevalence of voice disorders was 3.5 per 1000, aphasia was 1.9 per 1000, and fluency disorder was 0.7 per 1000 [37]. Furthermore, Sozda and Cole reported that visual learning and memory abilities were the same compared to verbal learning and memory performance in a sample of 103 veterans (14% Hispanic), verbal learning (SD = 1.1 below the mean) and verbal memory (SD = 1.4 below the mean) [38].

Based on behavioral and emotional results, Afari *et al.* found that among 554 OEF/OIF veterans (31.3% Hispanic), female veterans had higher rates of military sexual trauma and male veterans had higher levels of alcohol consumption [41]. However, TBI emotional (depression, aggression) and physical (pain intensity) symptoms were alike in males and females who experienced similar deployments. Furthermore, Rodgers *et al*. reported that from a sample of 310 (1.3% Hispanic) veterans with TBI, the mean of self-reported depression was 21.20 (STD = 11.76, range = 0–51) [42].

Post-traumatic stress disorder (PTSD) is a comorbidity that is strongly associated with TBI. A brief 2014 report by Olson-Madden, Forster, Huggins and Schneider used the After-Discharge Longitudinal Registry as a sampling frame. Thirteen percent of the 4391 veterans who participated were Hispanic [43]. After adjusting for covariates, the study found that current depressive symptoms (Combined RR = 1.09, 95% CI = 1.07–1.11) and PTSD (Combined RR = 2.00, 95% CI = 1.41–2.83) were significantly associated with the current suicidal ideation. Similarly, substance abuse studies revealed that 55% of veterans who were seeking treatment for substance abuse screened positive for TBI [44].

Descriptive epidemiological studies failed to distinguish a number of veterans with TBI, and most studies failed to identify individuals in need of care or primary sources of TBI [40]. Without this information, they were unable to identify specific targets for intervention. Additionally, cross-sectional studies that focused on mild TBI revealed risk indicators associated with the pathology of PTSD [37,38,39,42]. Sample sizes were small and most studies used a convenience sample of veterans who served specifically in missions in Iraq, Afghanistan, and similar geographic areas. There is consequently very little that can be definitively determined from these studies. Furthermore, because many of TBIs symptoms are similar to those of other disorders (e.g., PTSD), it is difficult to assess the severity of symptoms which can be attributed exclusively to TBI.

**Table 2:** Review on traumatic brain injury in United States Operation Enduring Freedom/Operation Iraqi Freedom (OEF/OIF) Hispanic veteran.

Study	Study Design, Population Sample	Sample Size (% Hispanic)	TBI Definition	Assessment	Outcomes	Critique
Patil *et al.* [40]	Retrospective cohort study Midwestern VA Polytrauma Network Site	246 (19%)	VA’s TBI screening. DoD TBI’s definition	Self-reported head pain occurring 30 days prior to initial MTBI screening. Headache severity: Neurobehavioral Symptoms Inventory (0 = none; 4 = almost always)	45% diagnosed with migraine headaches. 20% diagnosed with chronic daily headaches. 92% reported some level of sleep disturbances. Treatment: Triptan (68%) and anticonvulsant (55%), and tricyclic (40%). Headache rating did not differ by ethnicity (Hispanic/Latino *vs*. non-Hispanic/Latino) or marital status (married *vs*. unmarried)	Small sample size. Self-report data (not a valid instrument) for headaches. Low follow up (missing chart information)
Ettenhofer *et al*. [39]	Retrospective, cross-sectional design. VA Greater Los Angeles Healthcare System, GLA Polytrauma Network.	57 (21.4%)	VA’s TBI screening. TBI’s definition. Based on LOC or PTA: 1 = mild without LOC or PTA; 2 = mild with LOC or PTA; 3 = moderate for LOC and/or PTA; 4 = severe for LOC > 24 h and/or PTA > 7 days	Medical chart review. Clinical interview. Self-report inventory of PCS (Neurobehavioral Symptom Inventory). Battery of neuropsychological test.	Post concussive symptoms = 34% of veterans self-reported (severe to very severe). Involvement in work and school (*p* = 0.45, *p* < 0.001). Housing insecurity (*p* = −0.34, *p* < 0.05). Clinical-rated DSM-IV GAF (*p* = 0.43, *p* < 0.01)	Small sample size. Generability: Only one Polytrauma Network. 89.5% individuals were diagnosed with PTSD. Other co-morbidities could affect the functional status (*i.e.*, pain, sleep disturbances, *etc*.)
Norman *et al*. [37]	Retrospective, cross-sectional design	303,716 (~12%)	VA’s TBI screening. DoD TBI’s definition Severe: Normal or abnormal imaging, LOC > 24 h; AOC > 24, PTA > 7 days. Moderate: Normal or abnormal imaging, LOC > 30 min and ≤ 24 h, AOC > 24 h, PTA > 1 day and < 7 days.Mild: Normal imaging; LOC from 0–30 min, AOC is up to 24 h and PTA is from 0–1 day and unclassifiable.	ICD-9-CM code for communications disorders (aphasia, voice disorders and, fluency)	Voice disorders (3.5 per 1000). Aphasia (1.9 per 1000). Fluency disorder (0.7 per 1000). TBI with aphasia (OR = 11.09–252.75, 95% CI = 8.78–441.52, *p* < 0.01). TBI with fluency disorders (OR = 3.58–10.41, 95% CI = 2.56–42.40, *p* < 0.01). TBI and voice disorders (OR = 1.5–6.61, 95% CI = 1.24–14.05, *p* < 0.01)	Diagnostic bias: some veterans were only diagnosed once with communication disorders. Diagnostic categories too broad. No clear mechanism of injury, could be associated with speech-language problems
Rodgers *et al*. [42]	Retrospective, cross-sectional design Veterans Affairs Outpatient Level 3 Polytrauma clinic	310 (1.3%)	DoD TBI’s definition. VA’s TBI screening. Self-report TBI	Self-report of Beck Depression Inventory II (BDI-II) Score: 0 = no symptoms to 3 = severe symptoms. Total score of 21 = moderate symptoms	Mean BDI-II total score = 21.20 (SD = 11.76; range = 0–51) CFA’s models: three-factor TBI, two factor psychiatric, three-factor substance abuse, and two-factor neurorehabilitation samples. Chi-square for all models were significant (*p* < 0.001). Three-factor substance abuse model: reliability = 0.79 to 0.88 (best fit).	Instrument (BDI-II) was demonstrated to have a good reliability and validity in previous studies.3. Generalizability (only veterans referred to outpatient level 3polytrauma clinic. Small sample size to use CFA’s models.
Sozda *et al*. [38]	Cross-sectional study design Veterans Affairs Outpatient Level 3 Polytrauma clinic	103 (14%)	DoD TBI’s definition VA’s TBI screening	Wechsler Adult Intelligence Scale (WAIS-III) Digit Span subset. Delis-Kaplan Executive Functioning System. Hopskins Verbal Learning Test. Brief Visuospatial Memory Test. Beck Depression Inventory. PTSD Checklist-Military Version	Non-normality of neuropsychological (Wilsoxon’s sign ranked tests to compare median z scores of interest, and Cohen’s r effect sizes; small = 0.1, medium= 0.3, large = 0.5). Visual learning and memory abilities = intact compared to verbal learning and memory performance = reduced. Verbal learning (SD = 1.1 below the mean) and verbal memory (SD = 1.4 below the mean)	All MTBI’s were self- reported during soldier’s active duty status. Lack of methodology while identification of included/excluded patients. Controlled environment setting, Causal relationship between psychiatric symptoms, MTBI, and neuropsychological performance.

## 4. Discussion

DoD Section 508 of the Rehabilitation Act of 1973 requires the federal agencies to develop, obtain, and maintain records, to enable federal employees and members of the public with disabilities to have access to and use of information and data [45]. In compliance with the DOD, the CDC proposed criteria for a common TBI definition across agencies. This included a standard definition of TBI symptomatology in terms of its physical, cognitive, behavioral, and emotional impact. Unfortunately, it did not move forward, and the challenge for successfully identifying TBI cases and severity in OEF/OIF veterans remains.

The greatest challenge is to promote consistency among federal agencies (CDC, DoD/VA, *etc*.) when identifying TBI. As previously stated, there are multiple definitions of TBI and ICD-9-CM codes related to TBI. Overall, the estimated number of veterans with specific ICD-9-CM codes can vary substantially across agencies.

Furthermore, it is critical to consider minority groups when conducting studies. All agencies report race and ethnicity differences in studies, but the samples of minority veterans are often small. These small samples result in large standard errors; therefore, differences between Hispanic and non-Hispanic veterans often remain unreported. Previous research presents variation across social, cultural, and economic indicators among minority groups, which can have an important impact on outcomes. Nevertheless, many of these groups remain underrepresented. No study in OEF/OIF veterans thoroughly evaluated the importance of culture in the assessment and treatment of TBI.

Further research and funding is needed to understand the socioeconomic impact of military deployment. Studies on cost effectiveness of health interventions should include a focus on mental health disorders.

In the last 10 years, DoD/VA has invested billions of dollars in research to implement services targeting OEF/OIF veterans, which is positive, but monitoring and evaluation of mental health services is deeply flawed without the true number of veterans who have TBI, since there is no clear definition of the disease. Additionally, future clinical studies will benefit from larger sample sizes with more ethnic diversity since the US has the world’s most diverse military. A priority for the future includes investing in research that involves minorities so that policymakers and program planners can target the most effective methods for TBI treatment and rehabilitation. The VA recently launched a large, 10-year longitudinal study to collect data on health risk behavior and health care utilization by OEF/OIF veterans, which is a positive step. Finally, investigations into the pathogenesis of mental health disorders, and predictors of relapse and recovery among OEF/OIF veterans should be conducted.

## 5. Conclusions

This review revealed a dearth of rigorous research on TBI among Hispanic veterans. Existing literature has found high rates of physical, cognitive, behavioral and emotional symptoms in Hispanic veterans who have suffered TBI. PTSD is strongly associated with TBI, which complicates diagnosis and analysis. Some literature suggests considerable differences between the long term effects of TBI in Hispanic and non-Hispanic veterans. Despite this, a lack of a consensus on proper detection of TBI as well as an insufficient amount of research dedicated to this group severely limits the scope of conclusions that can be reached in this review.

Veteran health care providers ought to collaboratively develop and implement a standard definition of TBI as well as a standard set of instruments to detect it. Future research must include an emphasis on Hispanic veterans and other minorities in order to better understand the prevalence and sequelae in this unique group. This will be critical to the development of effective and targeted interventions.

## Appendix A: Search strategy

### MeSH Headings:

- Hispanic: Cuban American, Cuban Americans, Hispanics, Latina, Latinas, Latino, Puerto Rican, Puerto Ricans, Spanish American, Spanish Americans
- Veteran: Veterans
- Traumatic Brain Injury: Acute Brain injury, brain injury, brain laceration, brain lacerations, brain trauma, contusion, contusions, encephalopathy, mild traumatic brain injury, post traumatic TBI

### PubMed (Full-Text):

Terms: ((((latino) OR hispanic) AND traumatic brain injury) OR TBI) AND veteran = 556 articles as 4 March 2015. All 556 articles were screened. From which:

■ Included = 556; duplicates = none; articles screened = 556; articles excluded (reviews, proceedings, posters, conferences, commentaries, interviews, case reports and dissertations) = 502
■ Included for eligibility = 54
■ Excluded by criteria = 50
  1. No OEF/OIF Hispanic veterans = 37
  2. Data was qualitative = 3
  3. Study was not conducted in an adult U.S. Veteran living in United States = 6
■ Included: 4 articles

(((“United States Department of Veterans Affairs” [Mesh]) OR (“Veterans” [Mesh] OR “Veterans Health” [Mesh] OR “Veterans Disability Claims” [Mesh] OR “Hospitals, Veterans” [Mesh] OR “Military Family” [Mesh]))) AND ((latino OR hispanic) AND (TBI OR traumatic brain injury)) on 23 January 2015.

### LEXISNEXIS: Accessed by the VA Librarian

Terms: Hispanics and TBI search assisted by the VA librarian

■ No table to import to Microsoft Access; manual entries
■ Included=13 articles as 26 January 2015; duplicates= 2 by PubMed; articles screened= 11; articles excluded (reviews, proceedings, posters, conferences, commentaries, interviews, case reports and dissertations)= 0
■ Included for eligibility= 11 articles
■ Excluded= 11
  1. No OEF/OIF Hispanic veterans= 9
  2. Data was qualitative= 0
  3. Study was not conducted in an adult U.S. Veteran living in United States= 2
■ Included = 0

### PsycINFO:

Terms: MJ military veterans AND MJ traumatic brain injury OR tbi AND MJ latinos AND Hispanic

■ Exported table to Microsoft Access to access inclusion/exclusion criteria.
■ Included = 171, duplicated = 21 by Refworks; articles screened = 150; articles excluded (reviews, proceedings, posters, conferences, commentaries, interviews, case reports and dissertations) = 0
■ Included for eligibility = 150
■ Excluded = 129
  1. No OEF/OIF Hispanic veterans = 120
  2. Data was qualitative = 9
  3. Study was not conducted in an adult U.S. Veteran living in United States = 0
■ Included for final review = 21

### PILOTS:

Terms: (traumatic brain injury or TBI) AND (hispanic OR latino) AND (veteran OR military veteran) as 2 March 2015

■ Included = 8 articles; duplicated = 2; articles screened = 6; articles excluded (reviews, proceedings, posters, conferences, commentaries, interviews, case reports and dissertations) = 0
■ Included for eligibility= 6
■ Excluded= 3
  1. No OEF/OIF Hispanic veterans = 3
  2. Data was qualitative = 0
  3. Study was not conducted in an adult U.S. Veteran living in United States = 0
■ Included for eligibility = 3

### EBSCO-CINAHL Plus:

Terms: MH Brain Injuries AND MH Hispanics EBSCO-CINAHL Plus combined:

■ Included = 95 articles; duplicated (Brain Injuries AND Hispanics AND Veterans) = 94; articles screened = 1; articles excluded (reviews, proceedings, posters, conferences, commentaries, interviews, case reports and dissertations) = 0
■ Included for eligibility= 1
■ Excluded: 1
  1. No OEF/OIF Hispanic veterans = 1
  2. Data was qualitative = 0
  3. Study was not conducted in an adult U.S. Veteran living in United States = 0
■ Included for eligibility: 0

### EMBASE:

Terms: “veteran”/exp OR veteran AND (“hispanic”/exp OR hispanic) AND traumatic AND (“brain”/exp OR brain) AND (“injury”/exp OR injury)

■ Included = 12 articles; duplicated = 5; articles screened = 7; articles excluded (reviews, proceedings, posters, conferences, commentaries, interviews, case reports and dissertations) = 0
■ Included for eligibility = 7
■ Excluded = 7
  1. No OEF/OIF Hispanic veterans = 3
  2. Data was qualitative = 3
  3. Study was not conducted in an adult U.S. Veteran living in United States = 1
■ Included for eligibility = 0

### AMSUS:

Terms: TBI AND veteran AND Hispanic

■ Manually entered records on Access database
■ Included = 20 articles; duplicated = 5 articles with LEXIS NEXIS; articles screened = 15; articles excluded (reviews, proceedings, posters, conferences, commentaries, interviews, case reports and dissertations) = 1
■ Included for eligibility = 14
■ Excluded = 8
  1. No OEF/OIF Hispanic veterans = 4
  2. Data was qualitative = 3
  3. Study was not conducted in an adult U.S. Veteran living in United States = 1
■ Included for final review = 6

### Automatic updates:

■ PubMed e-Alerts request on 18 January 2015
■ ESCO alert on 4 March 2015
■ VACO Library Literature e-Alerts Request as 27 January 2015, 3:47 PM
■ Daily News Alert: Veteran/Military Suicide
■ Weekly PubMed/MEDLINE Alerts: OEF/OIF Veterans, Traumatic Brain Injury o Biweekly PubMed/MEDLINE Alert: Minority Veterans

## References

[1] Menon D.K., Schwab K., Wright D.W., Mass A.I. (2010). Position statement: Definition of traumatic brain injury. Arch. Psyc. Med. Rehabil..

[2] Institute of Medicine (2010). Returning Home from Iraq and Afghanistan: Preliminary Assessment of Readjustment Needs of Veterans, Service Members, and Their Families.

[3] Center for Military Health Policy Research (2008). Invisible Wounds of War: Psychological and Cognitive Injuries, Their Consequences, and Services to Assist Recovery. http://www.rand.org/content/dam/rand/pubs/monographs/2008/RAND_MG720.pdf.

[4] Bazarian J.J., Blyth B., Mookerjee S., He H., McDermott M.P. (2010). Sex differences in outcome after mild traumatic brain injury. J. Neurotrauma.

[5] Kirkness C.J., Burr R.L., Mitchell P.H., Newell D.W. (2004). Is there a sex difference in the course following traumatic brain injury?. Biol. Research Nurs..

[6] Arango-Lasprilla J.C., Rosenthal M., Deluca J., Komaroff E., Sherer M., Cifu D., Hanks R. (2007). Traumatic brain injury and functional outcomes: Does minority status matter?. Brain Inj..

[7] Arango-Lasprilla J.C., Ketchum J.M., Gary K.W., Kreutzer J.S., O’Neil-Pirozzi T.M., Wehman P., Marquez de la Plata C., Jha A. (2009). The influence of minority status on job stability after traumatic brain injury. PM&R.

[8] Gary K.W., Arango-Lasprilla J.C., Ketchum J.M., Kreutzer J.S., Copolillo A., Novack T.A., Jha A. (2009). Racial differences in employment outcome after traumatic brain injury at 1, 2, and 5 years post-injury. Arch. Phys. Med. Rehabil..

[9] Whitaker V.B., Ward-Murray E.M. (2008). Decreasing the gap in health care disparities research using novel concepts on time, trustworthiness, and education as methodological strategies. Insight.

[10] National Institute of Minority Health and Health Disparities (NIMHD) About NIMHD. http://www.nimhd.nih.gov/about/nimhdHistory.html.

[11] National Institute of Health (NIH) (2000). Public Law 106-525-NOV 22, 2000. http://history.nih.gov/research/downloads/PL106-525.pdf.

[12] Centers for Disease Control and Prevention (2015). CDC Health Disparities and Inequalities Report (CHDIR). http://www.cdc.gov/TraumaticBrainInjury.

[13] Centers for Disease Control and Prevention (2011). CDC Health disparities and inequalities report—United States, 2011. MMWR Morb. Mortal. Wkly. Rep..

[14] Egede L.E., Dismuke C., Echols C. (2012). Racial/ethnic disparities in mortality risk among US veterans with traumatic brain injury. Am. J. Public Health.

[15] Donnelly K.T., Donnelly J.P., Dunnam M., Warner G.C., Kittleson C.J., Constance J.E., Bradshaw C.B., Alt M. (2011). Reliability, sensitivity, and specificity of the VA Traumatic Brain Injury Screening Tool. J. Head Trauma Rehabil..

[16] Kelly D.F., Becker D.P. (2001). Advances in management of neurosurgical trauma: USA and Canada. World J. Surg..

[17] Fakhry S.M., Trask A.L., Waller M.A., Watts D.D. (2004). Management of brain-injured patients by an evidence-based medicine protocol improves outcomes and decreases hospital charges. J. Trauma.

[18] Centers for Disease Control and Prevention (2015). Traumatic Brain Injury in the United States: Fact Sheet. http://www.cdc.gov/traumaticbraininjury/get_the_facts.html.

[19] Terrio H., Brenner L.A., Ivins B.J., Cho J.M., Helmick K., Schwab K., Scally K., Bretthauer R., Warden D. (2009). Traumatic brain injury screening: Preliminary findings in a US Army brigade combat team. J. Head Trauma Rehabil..

[20] Hoge C.W., Goldberg H.M., Castro C.A. (2009). Care of war veterans with mild traumatic brain injury—Flawed perspectives. N. Engl. J. Med..

[21] Tanielian T., Jaycox L.H. (2008). Invisible Wounds of War: Psychological and Cognitive Injuries, their Consequences, and Services to Assist Recovery.

[22] Schneiderman A.I., Braver E.R., Kang H.K. (2008). Understanding sequelae of injury mechanisms and mild traumatic brain injury incurred during the conflicts in Iraq and Afghanistan: Persistent postconcussive symptoms and posttraumatic stress disorder. Am. J. Epidemiol..

[23] Evans C.T., St. Andre J.R., Pape T.L., Steiner M.L., Stroupe K.T., Hogan T.P., Weaver F.M., Smith B.M. (2013). An evaluation of the Veterans Affairs traumatic brain injury screening process among Operation Enduring Freedom and/or Operation Iraqi Freedom veterans. PM&R.

[24] Veterans Health Initiative (2010). Traumatic Brain Injury. http://www.publichealth.va.gov/docs/vhi/traumatic-brain-injury-vhi.pdf.

[25] Defense and Veterans Brain Injury Center (DVIC) (2015). DoD Worldwide Numbers for TBI. http://dvbic.dcoe.mil/dod-worldwide-numbers-tbi.

[26] Jaffee M.S., Meyer K.S. (2009). A brief overview of traumatic brain injury (TBI) and posttraumatic stress disorder (PTSD) within the Department of Defense. Clin. Neuropsychol..

[27] Bilmes L.J., Stiglitz J.E. (2008). The three Trillion Dollar War: The True Cost of the Iraq Conflict.

[28] USA Today (2008). VA to Increase Benefits from Mild Brain Trauma. http://usatoday30.usatoday.com/news/military/2008-09-22-tbibenefits_N.htm.

[29] Military.com Traumatic Brain Injury Overview. http://www.military.com/benefits/veterans-health-care/traumatic-brain-injury-overview.html.

[30] National Institute of Neurological Disorders and Stroke (2015). NINDS Traumatic Brain Injury Information Page. http://www.ninds.nih.gov/disorders/tbi/tbi.htm.

[31] Centers for Disease Control and Prevention (2015). Basic Information about Traumatic Brain Injury and Concussion. http://www.cdc.gov/traumaticbraininjury/basics.html.

[32] Defense and Veterans Brain Injury Center (DVIC) TBI Basics. http://dvbic.dcoe.mil/about-traumatic-brain-injury/article/tbi-basics.

[33] Veterans Health Administration (2010). Screening and Evaluation of Possible Traumatic Brain Injury in Operation Enduring Freedom (OEF) and Operation Iraqi Freedom (OIF) Veterans. http://www.va.gov/optometry/docs/vha_directive_2010-012_screening_and_evaluation_of_possible_tbi_in_oef-oif_veterans.pdf.

[34] Sayer N.A., Nelson D., Nugent S. (2011). Evaluation of the Veterans Health Administration traumatic brain injury screening program in the upper Midwest. J. Head Trauma Rehabil..

[35] Veteran Health Administration (2012). Report on VA Facility Specific Operation Enduring Freedom, Operation Iraqi Freedom, and Operation New Dawn Veterans Coded with Potential PTSD, from 1st Qtr FY 2002 through 3rd Qtr FY 2012. http://www.publichealth.va.gov/docs/epidemiology/ptsd-report-fy2012-qtr3.pdf.

[36] Liberati A., Altman D.G., Tetzlaff J., Mulrow C., Gøtzsche P.C., Ioannidis J.P.A., Clarke M., Devereaux P.J., Kleijnen J., Moher D. (2009). The PRISMA statement for reporting systematic reviews and meta-analyses of studies that evaluate healthcare interventions: Explanation and elaboration. BMJ.

[37] Norman R.S., Jaramillo C.A., Amuan M., Wells M.A., Eapen B.C., Pugh M.J. (2013). Traumatic brain injury in veterans of the wars in Iraq and Afghanistan: Communication disorders stratified by severity of brain injury. Brain Inj..

[38] Sozda C.N., Muir J.J., Springer U.S., Partovi D., Cole M.A. (2014). Differential learning and memory performance in OEF/OIF veterans for verbal and visual material. Neuropsychology.

[39] Ettenhofer M.L., Melrose R.J., Delawalla Z., Castellon S.A., Okonek A. (2012). Correlates of functional status among OEF-OIF veterans with a history of traumatic brain injury. Mil. Med..

[40] Patil V.K., St. Andre J.R., Crisan E., Smith B.M., Evans C.T., Steiner M.L., Pape T.L. (2011). Prevalence and treatment of headaches in veterans with mild traumatic brain injury. Headache.

[41] Afari N., Pittman J., Floto E., Owen L., Buttner M., Hossain N., Baker D.G., Lindamer L., Lohr J.B. (2015). Differential impact of combat on post-deployment symptoms in female and male veterans of Iraq and Afghanistan. Mil. Med..

[42] Rodgers M., Asaria M., Walker S., McMillan D., Lucock M., Harden M., Palmer S., Eastwood A. (2012). The clinical effectiveness and cost-effectiveness of low intensity psychological interventions for the secondary prevention of relapse after depression: A systematic review. Health Technol. Assess..

[43] Wisco B.E., Marx B.P., Holowka D.W., Vasterling J.J., Han S.C., Chen M.S., Gradus J.L., Nock M.K., Rosen R.C., Keane T.M. (2014). Traumatic brain injury, PTSD, and current suicidal ideation among Iraq and Afghanistan U. S. veterans. J. Trauma. Stress.

[44] Olson-Madden J.H., Forster J.E., Huggins J., Schneider A. (2012). Psychiatric diagnoses, mental health utilization, high-risk behaviors, and self-directed violence among veterans with comorbid history of traumatic brain injury and substance use disorders. J. Head Trauma Rehabil..

[45] Department of Defense (DoD) Section 508 Fact Sheet. http://dodcio.defense.gov/Portals/0/Documents/Section%20508/DoDS508_FactSheet_Compliant.pdf.

